# The development of opioid vaccines as a novel strategy for the treatment of opioid use disorder and overdose prevention

**DOI:** 10.1093/ijnp/pyaf005

**Published:** 2025-01-20

**Authors:** Mustafa Tuncturk, Shikha Kushwaha, Robin M Heider, Tyler Oesterle, Richard Weinshilboum, Ming-Fen Ho

**Affiliations:** Department of Psychiatry and Psychology, Mayo Clinic, Rochester, MN, United States; Department of Psychiatry and Psychology, Mayo Clinic, Rochester, MN, United States; Department of Psychiatry and Psychology, Mayo Clinic, Rochester, MN, United States; Department of Psychiatry and Psychology, Mayo Clinic, Rochester, MN, United States; Department of Molecular Pharmacology and Experimental Therapeutics, Mayo Clinic, Rochester, MN, United States; Department of Psychiatry and Psychology, Mayo Clinic, Rochester, MN, United States; Department of Molecular Pharmacology and Experimental Therapeutics, Mayo Clinic, Rochester, MN, United States

**Keywords:** opioid use disorder, vaccine therapy, immunopharmacotherapy

## Abstract

Opioid use disorder (OUD) affects over 40 million people worldwide, creating significant social and economic burdens. Medication for opioid use disorder (MOUD) is often considered the primary treatment approach for OUD. MOUD, including methadone, buprenorphine, and naltrexone, is effective for some, but its benefits may be limited by poor adherence to treatment recommendations. Immunopharmacotherapy offers an innovative approach by using vaccines to generate antibodies that neutralize opioids, blocking them from crossing the blood-brain barrier and reducing their psychoactive effects. To date, only 3 clinical trials for opioid vaccines have been published. While these studies demonstrated the potential of opioid vaccines for relapse prevention, there is currently no standardized protocol for evaluating their effectiveness. We have reviewed recent preclinical studies that demonstrated the efficacy of vaccines targeting opioids, including heroin, morphine, oxycodone, hydrocodone, and fentanyl. These studies showed that vaccines against opioids reduced drug reinforcement, decreased opioid-induced antinociception, and increased survival rates against lethal opioid doses. These studies also demonstrated the importance of vaccine formulation and the use of adjuvants in enhancing antibody production and specificity. Finally, we highlighted the strengths and concerns associated with the opioid vaccine treatment, including ethical considerations.

## IMMUNOPHARMACOTHERAPY FOR OPIOID USE DISORDER

Opioid use disorder (OUD) impacts over 40 million individuals globally and gives rise to a high economic burden on society.^1,2^ Medication for opioid use disorder (MOUD) is often considered the primary treatment approach for OUD.^3,4^ The US Food and Drug Administration (FDA) has approved 3 medications for the treatment of OUD, including methadone, buprenorphine, and naltrexone.^5,6^ In addition, naloxone, a μ-opioid receptor antagonist, can be used to reverse overdose effects.^7,8^ Moreover, nalmefene, an opioid receptor antagonist, was recently approved by the FDA for the treatment of opioid overdose.^9^ Current treatments for OUD are effective. However, many patients with OUD return to opioid use at some point within their lifetime. Staying in treatment significantly reduces the risks of overdose or opioid-involved overdose death. Therefore, an opioid vaccine could serve as a valuable complement to existing treatments for OUD, enhancing long-term recovery efforts. Despite receiving MOUD, the relapse rate during drug therapy can reach approximately 90% within a 1-year period.^10-12^ Immunopharmacotherapies emerged as a prospective treatment strategy for OUD as early as the 1970s.^13,14^ In 1974, Bonese et al. demonstrated the potential of active immunization by using a morphine-based hapten conjugated to bovine serum albumin (BSA), resulting in antibodies that reduced intravenous heroin self-administration in non-human primates.^15^ Although the concept of anti-opioid vaccines was introduced in the 1970s, interest in opioid vaccines increased significantly during the 1990s. This renewed interest was driven by the initiation of clinical trials for vaccines targeting cocaine and nicotine.^16-18^

Immunopharmacotherapies for OUD primarily involve monoclonal antibodies and opioid vaccines.^19,20^ Monoclonal antibodies provide rapid, reversible protection by directly neutralizing opioids in the bloodstream, making them suitable for acute overdose scenarios or when opioid pain relief is needed.^21,22^ A recent preclinical study demonstrated that monoclonal antibodies against the fentanyl class of drugs including fentanyl and carfentanil reduced the effects of both drugs in a dose-responsive manner in mouse antinociception models.^23^ Another preclinical study reported that monoclonal antibodies reversed fentanyl-induced antinociception, respiratory depression, and bradycardia, and that rats retained protection against additional challenges for at least 1 week.^24^ Nevertheless, monoclonal antibodies require frequent dosing and are costly, limiting their accessibility.^21,23^ Vaccines, in contrast, induce a long-lasting immune response that blocks opioids from reaching the brain, offering a more cost-effective and sustained prevention option. However, their prolonged action may pose challenges if patients need opioid-based pain management. By treating with opioid antigens, the vaccines prompt the production of antibodies that specifically bind to opioids, preventing their passage across the blood-brain barrier and subsequent psychoactive effects.^14,17^ Specifically, opioids are rapidly metabolized into multiple active compounds, which can be specifically targeted by vaccines to block their metabolites from crossing the blood-brain barrier. By preventing the formation of active metabolites, these vaccines may also reduce craving and compulsive use.^16,25^ Most studies of substance use disorder vaccines involve psychoactive substances such as nicotine, cocaine, oxycodone, heroin, or fentanyl—compounds that have low intrinsic immunogenicity. Thus, they are often conjugated to immunogenic proteins, and the opioids themselves function as haptens to stimulate antibody production.^26-28^ Commonly used protein conjugates include adenovirus capsid proteins, keyhole limpet hemocyanin, and tetanus toxoid.^29,30^ Preclinical animal studies play a pivotal role in demonstrating the efficacy and safety profiles of vaccines designed for the treatment of OUD.^14,31^ Those studies are typically conducted in rodent models and involve administering opioid vaccines to evaluate their capacity to elicit a robust immune response and mitigate the behavioral and neurobiological effects associated with opioid exposure. The outcomes of these preclinical investigations inform the rational design and optimization of vaccine formulation, dosage regimens, and adjuvant strategies, thereby guiding the selection of potential candidates for clinical trials.^17,18,31^

## PRECLINICAL AND CLINICAL VACCINE STUDIES: TARGETING HEROIN AND MORPHINE

Heroin is a schedule 1 substance under the Controlled Substances Act. Notably, approximately 727 000 individuals died from opioid overdoses between 1999 and 2022, with the rise in deaths occurring in 3 distinct waves. The first wave began in the 1990s with the increased prescribing of opioids, including natural and semi-synthetic opioids and methadone, although these deaths have decreased in recent years. The second wave began in 2010, marked by a sharp rise in overdose deaths related to heroin. However, recent years have seen a decline in heroin-related overdose fatalities. The third wave began in 2013, with a significant increase in overdose deaths involving synthetic opioids, especially those linked to illegally manufactured fentanyl and its analogs.^32-34^ The development of a vaccine against heroin is challenging because of the complexity of heroin metabolism and the rapid generation of active metabolites (ie, 6-acetylmorphine (6-AM), and morphine).^35,36^ Heroin is rapidly converted to 6-AM and subsequently transformed into morphine, and this is an imperative for vaccine design.^35-37^ Successful vaccine development hinges on the meticulous design of haptens, particularly targeting 6-AM, the principal psychoactive compound.^17^ The pioneering studies in the 1970s led by Wainer and Berkowitz laid the groundwork for morphine vaccine development,^38-40^ providing foundational insight into the production of morphine-specific antibodies. Immunization produces antibodies,^38,40,41^ which in turn alter neurobehavioral phenotypes induced by heroin, such as reduced antinociceptive effects^42^ and suppression of hypermotor activity.^41^ Furthermore, immunization against morphine with high-avidity antibodies reduces the concentration of free, metabolically active compounds in the serum, as well as their pharmacological effects.^39,40^ Notably, immunization of chicken eggs with a morphine vaccine showed no significant adverse effects on growth or development,^43^ demonstrating the potential safety of this vaccine. These findings from preclinical studies underscore the complex interplay between immunization, physiological responses, and pharmacological outcomes in modulating morphine effects (Table S1).

Effective vaccination impedes the distribution of heroin and its metabolites, particularly 6-AM, to the brain.^37,44-46^ This inhibition plays a crucial role in mitigating the reinforcing effects of heroin and its compulsive self-administration, characterized by a persistent and uncontrollable pattern of use.^37,45,47^ These vaccines demonstrate effectiveness in preclinical models by reducing (1) heroin-induced antinociception,^44,48,49^ (2) heroin-induced locomotor activity,^44,50,51^ and (3) the acquisition of heroin-induced conditioned place preference.^37^ Additionally, the selective inhibition of heroin-related behaviors was observed while allowing the concurrent use of opioid analgesics such as codeine, buprenorphine, and methadone.^37^ The endpoints of these preclinical studies underscore the importance of vaccine formulations and adjuvants in influencing antibody titers and specificity.^52,53^ Specifically, liposomes containing monophosphoryl lipid A and certain haptens such as DiAmHap and 6-PrOxyHap demonstrate their efficacy in enhancing antibody production and specificity.^52-54^ Furthermore, formulations with adjuvants, such as Toll-like receptor (TLR) agonists (TLR3 or TLR9) and alum prove to be robust inducers of anti-heroin antibody titers, protecting against heroin-induced antinociception.^55^ Moreover, Bremer et al. reported that their heroin vaccine elicited high titers of heroin-specific antibodies, leading to a significant reduction in the potency of heroin, an effect which was sustained after 3 rounds of vaccinations and persisted for over 8 months.^56^ Several studies also highlight the specificity of induced antibodies, with selectivity for heroin, 6-AM, and morphine, without cross-reactivity with other prescription opioids.^57,58^ Notably, regioselective deuteration of a heroin-hapten (HdAc) has been shown to enhance vaccine efficacy in blocking heroin-induced antinociception, demonstrating the importance of hapten structure.^49^ The combination of the vaccine with naloxone prolongs the antagonism of morphine’s effects, suggesting potential synergy between vaccines and other interventions.^59^ Collectively, these preclinical studies demonstrate the potential therapeutic value of heroin vaccination in the treatment of OUD.^47,54,60,61^

Unlike anti-nicotine and anti-cocaine vaccines, there is limited clinical data on opioid-specific vaccines, with only 3 published human clinical trials to date.^62-64^ In 2007, Akbarzadeh et al. reported a randomized double-blind placebo-controlled study, with 102 outpatient participants with OUD.^64^ The primary outcome of that trial demonstrated the safety and immunogenicity of a morphine vaccine. Patients received different doses of the morphine vaccine (12.5, 100, and 600 μg/mL). Specifically, participants received 3 intramuscular injections at day 0, day 30, and day 60 and were monitored for safety and antibody production over 12 months. All 102 volunteers completed the 3-injection course, with antibody levels monitored at 5, 7, 9, 11, and 12 months. The vaccine demonstrated good tolerability, dose-dependent increases in antibody levels, and no serious adverse events. Local pain and tenderness at the injection site were the most common side effects. Anti-morphine antibodies were detected after the first and second injections, reaching peak levels at 3 months and persisting for at least 1 year. This study demonstrated that morphine vaccines were well-tolerated, induced robust antibody responses, and could provide an alternative strategy for preventing relapse.^64^

In 2009, the same group conducted another trial involving 347 individuals with OUD, exploring the tolerability, safety, and efficacy of the same morphine vaccine at a single dose (50 μg/mL), which was administered 3 times on days 0, 30, and 60.^63^ The vaccine was well-tolerated and had fewer local side effects compared with other viral and bacterial vaccines. The enhancement was observed in anti-morphine antibody, total protein, and gamma globulin levels after the second and third vaccinations, peaking at 3 months but starting to decline by the fourth month. As anticipated, levels of anti-morphine antibodies were higher than baseline levels, even 1-year post-immunization. This study recommended re-vaccination within the first year.^63^ However, the clinical significance of these findings remains uncertain, as treatment-related outcomes and efficacy data are absent, leaving unanswered critical questions regarding the optimal dosing regimen and vaccine effectiveness.^62,63^

In 2012, Farhangi et al. reported the synthesis and efficacy of a morphine vaccine through the conjugation of morphine-6-hemisuccinate with BSA.^62^ A cohort of 436 individuals with OUD received a regimen of 3 doses of the morphine vaccine administered over a 60-day period. Anti-morphine antibody levels in participants were measured on the 90th and 360th days. This morphine vaccine exhibited a sustained ability to prevent relapse. However, a decrease in anti-morphine antibody levels was observed 1 year after vaccination. This study emphasized the safety and efficacy of morphine vaccines, highlighting their favorable profile with fewer adverse effects than vaccines for many infectious diseases.^62^ Notably, approximately 90% of the patients with OUD who completed the study maintained sobriety.^62^ Despite the favorable outcomes observed in these studies, there remains a scarcity of efficacy data. Additionally, BSA is a major beef allergen and has the potential to cause allergic reactions, thereby limiting widespread, long-term use, or repeated exposures.^65-67^ More comprehensive research is needed regarding the optimal dosing regimen, subsequent relapse rates, and the effective range of the antibody titers.^62,64^ Thus, future research is required to elucidate the safety, efficacy, and long-term impact of opioid vaccines in the treatment landscape of OUD.

## PRECLINICAL VACCINE STUDIES: TARGETING OXYCODONE AND HYDROCODONE

The overprescription of opioid analgesics in the United States has reached epidemic levels, posing a serious public health crisis.^68,69^ In the United States, about 3% of individuals aged 12 or older (approximately 8.6 million people) misused prescription pain relievers in the past year, according to 2023 data.^34^. Hydrocodone and oxycodone were the most misused prescription opioid pain relievers, accounting for 74.1% of misuse, while prescription fentanyl was misused by only 5.6% of individuals. Oxycodone was initially introduced to treat severe pain. However, its widespread availability and high abuse potential have led to a concerning trend of misuse and an increasing incidence of OUD, with many individuals transitioning to even more potent substances, such as fentanyl or heroin.^70,71^ In 2018, the global usage of oxycodone, including both prescribed and illegal forms, hit a peak of 45 717 defined daily doses.^69^ This level of consumption positioned oxycodone as the second most commonly used opioid, following fentanyl.^69^ Moreover, the United States accounted for approximately 73% of the global consumption of oxycodone between 1996 and 2016, highlighting its extensive use.^72^

The preclinical opioid vaccine studies targeting oxycodone have shown positive outcomes, suggesting the potential efficacy and safety of vaccination in combating OUD (Table S2). Several studies have demonstrated that the oxycodone vaccine effectively diminishes the reinforcing effects of oxycodone,^73-75^ and significantly decreases the distribution of opioids to the brain, potentially mitigating their central effects.^74,76-81^ Previous preclinical studies have highlighted a reduction in opioid-induced antinociception.^74-76,82^ Specifically, these studies reported that high-titer antibodies specific for oxycodone were produced after vaccination.^75,76,83-87^ Vaccine-related toxicity or adverse effects were not observed,^85^ underscoring the safety and tolerability of the vaccine. Several studies suggest that the opioid vaccine may protect against overdose and fatality by reducing the opioid distribution to the brain and its subsequent effects.^74,77,78,88,89^ Vaccine efficacy was influenced by the dosage and administration route, with improved outcomes observed after subcutaneous administration compared to intravenous delivery.^90^ Common outcomes evaluated in studies targeting hydrocodone vaccines include the decrease of antinociceptive efficacy for both hydrocodone and oxycodone, as well as potential cross-reactivity in serum antibodies. These studies also reported sustained efficacy in attenuating opioid antinociceptive effects and improved survival rates against lethal doses of both opioids.^77,89^

## PRECLINICAL VACCINE STUDIES: TARGETING FENTANYL, SYNTHETIC FENTANYL ANALOGS AND SYNTHETIC OPIOIDS

The US Centers for Disease Control and Prevention (CDC) reports that the United States is currently experiencing a “fourth wave” of opioid overdose deaths driven primarily by potent synthetic opioids , that is, fentanyl, carfentanil, and other synthetic fentanyl analogs.^32^ Carfentanil is a synthetic opioid. Its presence in illicit drug markets in the United States is alarming, as the potency of this drug could lead to an increase in overdoses and overdose-related deaths. Carfentanil is used as a sedative agent for elephants and other large mammals. Carfentanil is approximately 10 000 times more potent than morphine and 100 times more potent than fentanyl, which can be lethal at the 2-milligram range.^91^ While deaths from heroin and prescription opioids have remained relatively stable, fatalities involving synthetic opioids like fentanyl and carfentanil have dramatically increased in recent years.^92,93^ These substances pose a unique challenge due to their high potency, rapid onset of action, and narrow therapeutic window, which may significantly increase the risk of overdose.^94,95^ Notably, fentanyl and its analogs have been implicated in the majority of fatal opioid overdoses, surpassing other opioids in their lethality.^96,97^ The CDC’s National Center for Health Statistics reported that opioid overdose deaths were estimated at 81 083 in 2023, with synthetic opioids, primarily fentanyl, associated with over 90% of these deaths.^94,95,98^ Fentanyl, originally developed for anesthesia and severe pain management, is now widely misused and has become a primary driver of opioid overdoses. Overdose due to illicit synthetic opioids (eg, fentanyl and fentanyl analogs) continues to rise.^32^ Illicitly manufactured fentanyl is often mixed with or sold as heroin. According to the Drug Enforcement Administration (DEA) laboratory testing, 6 out of 10 fentanyl-laced counterfeit prescription pills now contain a potentially lethal dose of fentanyl.^99^

The potency of fentanyl is approximately 50-100 times greater than that of morphine, which significantly increases the risk of overdose due to its ability to induce respiratory depression at much lower doses.^100,101^ Carfentanil, a synthetic analog of fentanyl, is about 100 times more potent than fentanyl, meaning it can exert its effects at even smaller doses, thereby dramatically increasing the risk of fatal overdose.^102^ This extreme potency of fentanyl and carfentanil poses a significant challenge for overdose management, as even a slight miscalculation in dosage can lead to lethal outcomes. The high potency of these synthetic opioids not only raises the risk of accidental overdose but may also affect the efficacy of therapeutic interventions like opioid vaccines. Vaccines targeting these opioids need to generate sufficiently high titers of antibodies to effectively neutralize these potent compounds before they can reach the brain and exert their effects. The surge in overdoses involving these synthetic opioids underscores the urgent need for innovative strategies to prevent and treat OUD and overdose.^103-105^

Several preclinical studies have investigated vaccines for both fentanyl and synthetic fentanyl analogs. The main outcome measures were (1) a reduction in potency and reinforcing effects,^106,107^ (2) levels of anti-fentanyl antibodies,^86,108-111^ (3) blocking of fentanyl and synthetic fentanyl analog-induced antinociception,^108,109,111-114^ and (4) respiratory depression^109,112-115^ (Table S3). These vaccines exhibited sustained efficacy over time^106^ and were effective in altering drug distribution to the brain, thereby reducing central nervous system effects.^87,96,114,116^ Some vaccines demonstrated efficacy comparable to traditional opioid antagonist medications like naltrexone,^106,107^ with the onset of vaccine action sometimes being slower but displaying prolonged duration compared to standard treatments.^117^ Additionally, the vaccines demonstrated high selectivity by not interfering with the pharmacological activity of other opioids such as oxycodone, heroin, or methadone.^113^ A recent vaccine preclinical trial against U-47700, a synthetic opioid, demonstrated the vaccine’s ability to alter drug distribution, leading to substantial drug retention in the bloodstream and reducing diffusion into the brain.^Park et al., 2023^ Benzimidazole opioids, also known as nitazenes, are a group of new psychoactive substances that have recently led to an increase in fatal overdoses in the United States and Europe.^119^ These opioids have emerged in the illicit drug market, posing a growing public health threat. A recent preclinical study of a vaccine targeting benzimidazole-derived new psychoactive substance opioids (BNO) demonstrated its efficacy in generating robust antibody responses against various BNO drugs.^120^ That vaccine also effectively mitigated opioid molecules crossing the blood-brain barrier, opioid-induced respiratory depression, and opioid-induced antinociception.^120^ These findings collectively underscore the potential of vaccines as a novel approach to mitigate the effects of fentanyl and its analogs.

## BIVALENT OPIOID VACCINES

The opioid crisis has also led to a concerning rise in poly-opioid use, particularly due to the addition of potent synthetic opioids, such as fentanyl to street heroin.^104,121-123^ This combination exacerbates respiratory depression and increases the risk of overdose and death. Recent studies have shown that fentanyl contamination of heroin can drastically increase the risk of overdose due to fentanyl’s high potency and its potential to induce severe respiratory depression, leading to enhanced brain hypoxia.^124^ Researchers are exploring bivalent vaccines, which contain structurally different immunoconjugates to target multiple opioids simultaneously. These vaccines exhibit promising efficacy in conferring protection against the use of multiple opioids, a prevalent contemporary challenge.^14^ With the surge in overdose cases linked to fentanyl-contaminated heroin, the development of multivalent vaccines may be an innovative approach to combating opioid overdose and OUD by targeting multiple opioids with distinct chemical structures.^14,17^ Since opioid users often engage in concurrent use of multiple opioids or transition between different opioids, a multivalent vaccine strategy might offer a more effective solution, potentially conferring long-lasting protection against overdose and relapse.^14,17,21,125^

Bivalent vaccines combining heroin and fentanyl antigens showed efficacy in reducing opioid-induced antinociception, indicating potential synergistic effects in preclinical studies^126-128^ (Table S4). These vaccines elicited potent antibody responses, effectively reducing the distribution of opioids in the brain, thus mitigating their central effects.^127,129-131^ The bivalent vaccine formulations exhibited strong binding capabilities against a range of opioids, including heroin, fentanyl, and oxycodone, as well as their mixtures, demonstrating broad-spectrum protection.^132-134^ In addition, lyophilized vaccine formulations have maintained their effectiveness for up to a year when stored at room temperature.^129^ It has been reported that a dual fentanyl/heroin vaccine did not affect the antinociceptive potency of methadone, highlighting the vaccine’s selective efficacy. This result suggests that combining methadone with the vaccine could be beneficial for treating OUD and managing pain.^132^ Overall, these findings underscore the possible value of bivalent vaccination strategies for mitigating the harmful effects of opioids, paving the way for further clinical development and evaluation. Currently, a phase I/II clinical trial (NCT04458545) is ongoing in the United States. This trial was designed to evaluate the safety and efficacy of reducing opioid use, the magnitude of antibody responses, and the efficacy of a bivalent vaccine targeting oxycodone and heroin.

## CONCLUSIONS AND FUTURE PERSPECTIVES

The development of vaccines against OUD presents a novel approach for addressing the challenges associated with existing treatment approaches. Opioid vaccines may be beneficial for some patients due to their capacity to provide prolonged protection, fewer side effects, high selectivity, and reduced misuse potential. Opioid vaccines do not target the FDA-approved pharmacotherapies, such as methadone, buprenorphine, and naltrexone. Thus, the concurrent use of these medications alongside the vaccine underscores its potential benefits. A key advantage of opioid vaccines is their selective effectiveness in blocking the effects of substances like fentanyl and heroin, while leaving medications that act on the μ-opioid receptor unaffected.

Although vaccine treatment studies for OUD have been known for more than 50 years, there is still no effective vaccine available for clinical implementation. Despite the promising aspects which we have outlined here, there are also challenges. First, patients with OUD generally do not use a single type of opioid, rather, they often use multiple opioids or other substances simultaneously, such as stimulants.^135^ The use of multiple substances at varying doses in heterogeneous cocktails raises questions regarding the appropriate vaccine choice. Second, the optimal schedule of inoculations, the recommended frequency of boosters, and the effective range of antibody titers all remain unanswered. Individual variability in antibody titers and the potential for substitution of different drugs pose challenges, especially for compounds with small molecular weights that may not be recognized by the immune system. Finally, individuals may choose different opioids that are not targeted by the vaccine to achieve the desired euphoric effects or try to override the protective effects of the vaccine by using higher doses of opioids. It should be noted that vaccines alone may not alleviate craving, which is one of the critical factors for relapse. Vaccines against opioids produce antibodies that neutralize opioids, preventing them from crossing the blood-brain barrier and diminishing their psychoactive effects. In addition to preventing relapse, vaccines could potentially save lives by offering protection in cases of relapse involving highly potent opioids. OUD has a neurobiological component that involves tolerance, withdrawal, and craving. However, it remains unclear whether opioid vaccines can influence these aspects. More human clinical research will be required to assess the safety, efficacy, and long-term impact of opioid vaccines. Future research will also need to address concerns with regard to the heterogeneity of immune responses, the implementation of multivalent strategies for both multi-opioid and diverse substance use, and the acceptance by diverse populations.

Furthermore, the ethical landscape surrounding opioid vaccines is intricate and multifaceted.^136^ Key concerns revolve around the voluntary vs mandatory use of vaccines, the prioritization of vulnerable and marginalized groups, and the ethical implications of prophylactic vaccination.^137-139^ Integrating opioid vaccines into existing treatment protocols necessitates careful management to mitigate unintended health disparities, particularly in scenarios involving pain management.^136,138^ Opioid vaccines typically stimulate a sustained antibody response by activating the immune system, with the potential for cross-reactivity among different opioids. However, most opioid vaccine studies emphasize selectivity as a primary objective. This raises ethical concerns regarding the duration of the vaccine’s effects, particularly if patients might later require opioid-based pain relief or emergency surgery. Weitzman et al., 2024 Furthermore, it is possible that the implementation of specific opioid-targeting vaccines, that is, heroin in patients with OUD, may inadvertently drive individuals toward more potent and dangerous substances like fentanyl analogs to achieve euphoric effects.^14,139^ It is an open question whether patients with immune deficiency-related diseases should be vaccinated. Given that long-term opioid use is associated with immune insufficiency, the effectiveness of vaccination is unclear. The ethical issue of involuntary administration raises significant concerns regarding personal autonomy and informed consent. That issue is particularly contentious if mandates are considered for individuals in the justice system, pregnant women, or parents involved in child welfare disputes.^137,138,141^ Ensuring the safety and efficacy of these interventions in voluntary settings is essential before contemplating their mandatory use. Ethical deliberations must carefully weigh the potential benefits of reducing relapse against the risks of violating individual rights and autonomy, underscoring the complexity of integrating opioid vaccines into public health strategies. These issues highlight the complexities and ethical nuances inherent in the development, deployment, and implementation of opioid vaccines.

In conclusion, while MOUD is effective, its benefits may be limited by poor adherence to treatment recommendations. Therefore, a complementary approach, such as vaccine-based treatment, could serve as an additional option during the ongoing opioid crisis. Immunotherapies arouse great interest as a “hot topic” in the treatment of many psychiatric diseases such as schizophrenia,^142^ depression,^143^ obsessive-compulsive disorder^144^ and autism.^145,146^ The use of vaccines to treat substance use disorders is one of the most dynamic and actively researched areas in the field. There is a clear potential that opioid vaccines could be a novel alternative treatment approach for OUD and overdose prevention. However, for the reasons mentioned above, it is highly unlikely that vaccine therapy will be a definitive cure or the sole treatment for OUD. Despite the challenges, opioid immunotherapy presents a potentially impactful treatment strategy during the ongoing opioid epidemic, offering potential benefits in overdose prevention and OUD treatment. However, extensive research and careful ethical consideration are needed to fully develop and implement this potential therapeutic approach.

## Supplementary Material

pyaf005_suppl_Supplementary_Table_S1

pyaf005_suppl_Supplementary_Table_S2

pyaf005_suppl_Supplementary_Table_S3

pyaf005_suppl_Supplementary_Table_S4

## Data Availability

No new data were generated or analyzed in support of this research.
