# Supplementary material for: The development of opioid vaccines as a novel strategy for the treatment of opioid use disorder and overdose prevention
Source: Int J Neuropsychopharmacol. 2025 Jan 20;28(2):pyaf005. doi: 10.1093/ijnp/pyaf005 (PMC11792077; doi:10.1093/ijnp/pyaf005)
Supplement: pyaf005_suppl_Supplementary_Table_S1 [file pyaf005_suppl_supplementary_table_s1.pdf]

Supplementary Table 1. Vaccines targeting heroin and morphine

References	Drugs	Animal	Main Findings	Vaccine types
(Schlosburg et al., 2013)	Heroin	Rats	• Blocked the distribution of heroin and its metabolite (6-AM) to the brain.<br>• Inhibited heroin- and morphine-induced place preference and prevented the re-escalation of compulsive heroin self-administration.<br>• Did not interfere with the efficacy of opioid analgesics such as codeine, buprenorphine, and methadone, permitting their concurrent use.	Her–KLH, [The carrier protein used in the vaccine is keyhole limpet hemocyanin (KLH)]
(Méndez et al., 2023)	Heroin	Mice	• Over a period of 14-16 weeks, five booster injections with the same unit dose and adjuvant were administered, followed by the collection of blood and serum 14 days after each injection.<br>• Stress induced by both drug withdrawal and immobilization resulted in a decreased heroin-specific antibody titer.<br>• Unable to attenuate heroin-induced antinociceptive effects and locomotor activity in stressed conditions.	The morphine-6-hemisuccinate-tetanus toxoid (M6-TT) vaccine
(Raleigh et al., 2013)	Heroin	Rats	• Significant reduction in 6-MAM and morphine distribution to the brain.<br>• Reduced heroin-induced analgesia and blocked heroin-induced locomotor activity.	M-KLH, (M stands for morphine, and KLH stands for keyhole limpet hemocyanin)
(Raleigh et al., 2014)	Heroin	Rats	• Effectively blocked heroin-primed reinstatement of heroin self-administration.<br>• Decreased self-administration at low heroin unit doses while leading to a compensatory increase at high unit doses.<br>• Altered heroin and metabolite distribution, reducing concentrations in plasma and the brain.	Morphine-conjugate vaccine (M-KLH)
(Matyas et al., 2013)	Heroin	Mice	• Immunized at weeks 0, 3, and 6, and serum IgG responses to the vaccine were measured nine weeks after the initial immunization.<br>• Mixing unconjugated , tetanus toxoid (TT) with L(MPLA) resulted in higher anti-TT titers compared to TT conjugates.<br>• Liposomes containing monophosphoryl lipid A (L(MPLA)) effectively induced antibodies to heroin hapten analogs, enhancing the development of a potential heroin vaccine.	L(MPLA+ HerHap-PEG–MPER carrier)" and "L(MPLA+ OMAHapPEG–MPER carrier)
(Matyas et al., 2014)	Heroin	Mice	• Antibodies induced by DiAmHap exhibited specific cross-reactivity with heroin, 6-acetylmorphine, and morphine.<br>• Immunized mice with DiAmHap and MorHap showed reduced antinociceptive effects caused by heroin.	A heroin-like hapten (DiAmHap) conjugated to tetanus toxoid and mixed with liposomes containing monophosphoryl lipid A

(Jalah et al., 2015)	Heroin	Mice	• Significantly enhanced vaccine-induced antinociceptive effects.<br>• Antibodies bound effectively to heroin and its metabolites, namely 6-acetylmorphine and morphine.<br>• Displayed higher affinities for 6-acetylmorphine and morphine.	A heroin-, tetanus toxoid conjugate (TT) mixed with liposomes containing monophosphoryl lipid A [L(MPLA)]
(Stowe et al., 2011)	Heroin	Rats	• Six immunizations were administered to rats on days 0, 14, 28, 53, 108, and 151, with titer levels for both vaccines consistently increasing and peaking after the third administration (second boost, t = 53 days).<br>• Achieved rapid and robust polyclonal antibody titers with high specificity against heroin, 6-acetylmorphine (6AM), and morphine.<br>• The heroin-like vaccine showed a reduced likelihood of acquiring heroin self-administration.<br>• Heroin-vaccinated rats maintained the ability to acquire lever-pressing behavior for a natural reward (sweetened water).<br>• Blocked the antinociceptive effects of heroin.	The heroin-like vaccine (Her-11b), the morphine-like vaccine (Mor-12b)
(Bremer et al., 2014)	Heroin	Mice	• Administered on days 0, 14, and 28, with the samples collected on days 21 and 42.<br>• Immunized with a heroin vaccine via intraperitoneal (ip) injection or ip/subcutaneous (sc) coadministration demonstrated superior anti-heroin titers compared to subcutaneous injection.<br>• Improved vaccine effectiveness against heroin-induced analgesia.<br>• CpG ODN 1826 addition to the vaccine significantly increased protection from heroin addiction.	Heroin–KLH conjugate, TLR9 agonist cytosine-guanine oligodeoxynucleotide 1826 (CpG ODN 1826)
(Bremer et al., 2017)	Heroin	Rhesus monkeys, mice	• Antibodies reduced the potency of heroin.<br>• Demonstrated durable effects, persisting for over eight months.<br>• The second and third rounds of vaccinations maintained efficacy at 50-70% of the initial level.	Heroin-tetanus toxoid (TT) conjugate formulated with adjuvants alum and CpG oligodeoxynucleotide (ODN)
(Bonese et al., 1974)	Heroin	Rhesus monkey	• Effectively blocked the physiological effects of heroin that contribute to maintaining self-administration behavior.<br>• Significantly reduced intravenous heroin self-administration.	Morphine-6-hemisuccinyl-bovine serum albumin (M-6-HS-BSA)
(Hwang et al., 2019)	Heroin	Mice	• The Her-Hsp70 conjugate induced anti-heroin antibody production and demonstrated efficacy in blunting heroin-induced antinociception.<br>• Induced serum antibodies, and effectively sequestering free heroin in the bloodstream.	Her-BSA, Her-KLH and Her-Hsp70
(Bremer and Janda, 2012)	Heroin	Mice	• Administered on days 0, 14, and 28, with serum collected on days 21 and 42, showing higher titers on day 42.<br>• The Th2 humoral response elicited by alum, is essential for its anti-heroin potency.	Vaccine containing a more hydrolytically stable hapten

			• CpG ODN had a significant negative effect on titer levels and antibody specificity, potentially reducing the overall humoral response against heroin.	analogue and a Th1 adjuvant (CpG ODN)
(Sulima et al., 2018)	Heroin	Mice and rats	• Effectively against heroin-induced antinociception and locomotor changes after subcutaneous and intravenous heroin challenges.<br>• Vaccinated mice exhibit a reduction in heroin-induced hyperlocomotion.	TT−1 and TT−3 conjugate vaccines were mixed with Army Liposome Formulation (ALF)
(Torres et al., 2017)	Heroin	Mice	• Both palm-CV2 and palm-LV2 antibodies strongly inhibited heroin-associated behavioral effects, including hyper-locomotion and antinociception.	Heroin-HIV-1 (H2) vaccine: A synthetic heroin analog (MorHap)
(Hwang et al., 2018)	Heroin	Mice	• A systematic exploration of 20 heroin conjugate vaccine formulations was conducted, varying carrier proteins and adjuvants.<br>• Mice were given subcutaneous immunizations on days 0, 14, and 28, and blood samples collected at weeks 6 and 10 showed that the highest titer levels occurred at week 6.<br>• Adjuvants included a Toll-like receptor 9 (TLR9) agonist (CpG ODN 1826) and a TLR3 agonist (virus-derived double-stranded RNA), both used with alum.<br>• Vaccine formulations containing either TLR3 or TLR9 agonists with alum elicited strong antiheroin antibody titers and blocked heroin-induced antinociception.<br>• Combining TLR3 and TLR9 adjuvants did not enhance vaccine efficacy.<br>• Stability testing showed the TLR9 formulation remained stable for 30 days when stored as a lyophilized solid or liquid, while the TLR3 formulation did not.<br>• Mice immunized with the TLR9 + alum vaccine were significantly protected from lethal heroin doses, indicating its suitability for mitigating heroin's harmful effects even after month-long room-temperature storage.	A formulation involving Toll-like receptor 9 (TLR9) agonist and alum (aluminum-containing adjuvant) in combination with a heroin conjugate vaccine
(Li et al., 2014)	Heroin	Mice	• Antibody titers were approximately 10,000 U/mL three weeks after primary immunization with each hapten, remaining stable at six weeks post-immunization, following vaccinations administered to mice at weeks 0 and 6.<br>• Immunization with the haptens induced high-titer antibodies against each hapten, with DiAmHap generating the highest titers.<br>• Competitive ELISA demonstrated antibody specificity, with antibodies from 6-PrOxyHap-immunized mice showing effective binding to heroin, 6-acetylmorphine, morphine, and codeine. DiAmHap-induced antibodies exhibited high affinity for binding to DiAmHap, leading to challenges in the competition ELISA.<br>• Immunization with 6-PrOxyHap and DiOxyHap resulted in reduced antinociceptive effects of injected heroin.	6-PrOxyHap, DiPrOxyHap and DiAmHap

(Belz et al., 2020a)	Heroin	Mice	• Regio-selective deuteration of a heroin-hapten (HdAc) demonstrated greater efficacy in blunting heroin analgesia compared to the non-deuterated counterpart (HAc) in murine behavioral models.<br>• Vaccinated on days 0, 14, 28, and 51, with blood samples collected on days 21, 42, and 71, and midpoint antibody titer results at week 11 were determined from crossover ELISA experiments.<br>• HdAc vaccine generated higher antibody titers and exhibited equivalent or higher affinity antibodies towards heroin and its psychoactive metabolites (6-AM) compared to the HAc vaccine.<br>• The HdAc vaccine outperformed the HAc vaccine in behavioral tests, specifically in antinociception hot plate and tail-flick assays.	HdAc–BSA and HAc–BSA
(Gutman et al., 2021)	Heroin	Mice	• Mice immunized with TT-3 showed partial but significant protection against heroin-induced antinociception.<br>• Sera from mice immunized with TT-1 and TT-3 significantly sequestered 6-acetylmorphine (6-AM).<br>• Antibodies induced by TT-1, TT-2, and TT-3 had distinct selectivity toward heroin, 6-AM, and morphine.<br>• Sera from mice immunized with TT-3 showed no cross-reactivity with therapeutic drugs (buprenorphine, methadone, naloxone, and naltrexone).	Heroin vaccines; TT-1, TT-2, and TT-3;<br>The linker attachment site at C14,<br>1= (6,14-AmidoHap), 2= (14-AmidoMorHap), and 3= (14-AmidoHerHap) as novel heroin haptens.
(Belz et al., 2020b)	Heroin	Mice	• The HMsAc vaccine showed the highest affinity for anti-heroin antibodies, followed by the HAc vaccine, and then the H(Ds)2 vaccine.<br>• The HAc vaccine sequestered a higher amount of the active metabolite 6-AM and demonstrated a lower brain-to-blood ratio compared to the HMsAc and H(Ds)2 vaccine groups.	The HMsAc vaccine, the HAc and H(Ds)2 vaccine
(Sulima et al., 2022)	Heroin	Mice	• Haptens 2 and 3 induced protective effects against heroin in vivo, while epimeric analogues (1 and 4) showed no protective efficacy.<br>• TT-2 and TT-3 provided significant protection against the antinociceptive effects of heroin.<br>• TT-2 and TT-3 induced antibodies capable of binding to heroin and its active metabolite, 6-acetyl morphine, with no cross-reactivity with morphine, methadone, naloxone, or naltrexone.	TT-1, TT-2, TT-3, and TT-4;<br>1= (1-AmidoMorHap),<br>2= (1-AmidoMorHap epimer),3= (1 Amido-DihydroMorHap), and 4= (1 AmidoDihydroMorHap epimer)
(Méndez et al., 2021)	Morphine and heroin	Mice	• Vaccine-generated antibodies reduced the antinociceptive effect of morphine doses below 5 mg/kg; however, they did not affect doses above 10 mg/kg.<br>• Selectively decreased antinociception induced by morphine.<br>• Did not alter the antinociceptive effects of tramadol, gabapentin, and fentanyl.<br>• Did not block naloxone's antagonistic effect on morphine-induced antinociception.	The morphine/heroin vaccine (M-TT)

			• Combination of vaccination and naloxone prolonged the antagonism of morphine's effects.	
(Li et al., 2015)	Morphine and Heroin	Rats	• The triggered antibodies demonstrated high specificity for morphine, 6-acetylmorphine, and heroin.<br>• Antibodies showed no interaction with dissimilar therapeutic opioid compounds, such as buprenorphine, naloxone, and nalorphine.<br>• Significantly inhibited the increase in dopamine levels in the nucleus accumbens.<br>• Inhibited morphine-induced locomotor sensitization and heroin-primed reinstatement into heroin-seeking behavior.	Morphine-(ε-trifluoroacetylcaproyloxy) TFCS- keyhole limpet hemocyanin (KLH)
(Li et al., 2011)	Morphine and heroin	Rats	• Rats received vaccine boosts on days 14, 28, and 42, with titers still reaching approximately 1:10000 sixty days after the final injection.<br>• Produced high antibody titers specific for morphine, displaying selectivity for both morphine and heroin.<br>• Decreased morphine-induced locomotor activity in rats after immunization.<br>• Decreased dopamine levels in the nucleus accumbens post-morphine administration, aligning with its observed behavioral effects.<br>• Effectively inhibited morphine-induced locomotor sensitization and heroin-seeking behavior, highlighting its efficacy in suppressing both the psychomotor and reinforcing effects of opioids.	Morphine–keyhole limpet hemocyanin (KLH)
(Anton and Leff, 2006)	Morphine and heroin	Male adult rats	• Over 14–16 weeks, the rats received 7–8 biweekly booster injections, and sera collected 14 days after each boost showed sustained maximal antibody titers after the fourth or fifth injection.<br>• Induced a robust and sustained humoral response, generating high titer anti-morphine antibodies with specificity for both morphine and heroin.<br>• Equivalent specificity for heroin and its metabolites, with high affinity.<br>• Effectively blocked the reinforcing effects of heroin.<br>• Blocked the reacquisition of heroin self-administration behavior.	Morphine-tetanus toxoid (M-TT) vaccine
(Kosten et al., 2013)	Morphine	Rats	• Reduced antinociceptive responses to morphine.<br>• Lower brain morphine levels.<br>• Reduction in morphine conditioned place preference.	KLH-6-SM, [a conjugate vaccine against morphine, where KLH stands for keyhole limpet hemocyanin, and 6-SM stands for 6-succinylmorphine.]
(Akbarzadeh et al., 1999)	Morphine	One male goat, Six male	• Produced antibody after 8 weeks.<br>• Active immunization suppressed mouse locomotor activity.<br>• Suppression of morphine-induced hypermotor effects.	Morphine-6-succinyl-BSA (M-6-S-BSA) hapten

		rabbits, Six male mice, Six male rats		
(Farhangi et al., 2010)	Morphine	Chicken eggs	• No significant changes in weight were observed between the groups.<br>• Both groups exhibited healthy, normal growth and evolution.<br>• The comparison of growth and evolution did not reveal any adverse reactions in eggs injected with the therapeutic human morphine vaccine.	Morphine vaccine, including morphine-6-succinyl-bovine serum albumin hapten
(Wainer et al., 1972)	Morphine	Rabbits	• Produced antibody production after 8 weeks.	Morphine-6-hemisuccinate conjugated to bovine serum albumin (BSA)
(Hill et al., 1975)	Morphine	Rabbits	• Morphine clearance rate significantly slowed down by 24 hours after injection.<br>• The final circulating half-life of morphine increased with higher antibody avidity.<br>• Antibodies bind morphine-3-glucuronide less efficiently than native morphine.	Morphine-6-hemisuccinate bovine serum albumin (M-6-HS-BSA)
(Berkowitz and Spector, 1972)	Morphine	Mice	• Developed serum antibodies capable of binding dihydromorphine.<br>• Diminished effects of morphine.<br>• Altered concentration of morphine in the plasma.	3-Carboxymethyl-morphine-BSA
(Farhangi et al., 2012)	Morphine	436 subjects with OUD	• A total of 436 subjects with OUD were immunized with the morphine vaccine, receiving three doses on days 0, 30, and 60.<br>• Each dose contained 50 µg morphine-6-succinate-BSA, 0.5 mg aluminum hydroxide (Al^{3+}), 8 mg sodium chloride, 1.12 mg dibasic sodium phosphate anhydrous, 1.1 mg monobasic sodium phosphate monohydrate, and 1 ml water for injection.<br>• Blood samples were collected before immunization, 90 days after the first dose, and from 10% of participants randomly selected one year post-immunization.<br>• Total protein, gamma globulin, and anti-morphine antibody levels decreased over time but did not return to baseline even one year after the last dose.<br>• Well-tolerated and effectively prevented vaccinated individuals from resuming morphine use by neutralizing consumed morphine<br>• About 90% of participants stayed off morphine after completing the study.	Morphine-6-hemisuccinate-BSA
(Akbarzadeh et al., 2009)	Morphine	347 subjects with OUD	• Concentration of anti-morphine antibody showed a direct correlation with the number of injections.<br>• Anti-morphine antibody levels peaked at three months after the first injection and remained stable above baseline for a year.<br>• Well-tolerated, with no serious adverse events.	Morphine-6-succinate-BSA

(Akbarzadeh et al., 2007)	Morphine	102 subjects with OUD	• Well-tolerated, with no severe drug-related side effects.<br>• Antibody levels stayed elevated above baseline for at least a year across all dosage groups.<br>• No adverse events were reported during the one-year follow-up period after vaccination.	Morphine-6-succinate-BSA
