# Supplementary material for: The development of opioid vaccines as a novel strategy for the treatment of opioid use disorder and overdose prevention
Source: Int J Neuropsychopharmacol. 2025 Jan 20;28(2):pyaf005. doi: 10.1093/ijnp/pyaf005 (PMC11792077; doi:10.1093/ijnp/pyaf005)
Supplement: pyaf005_suppl_Supplementary_Table_S2 [file pyaf005_suppl_supplementary_table_s2.docx]

| **Supplementary Table 2.** Vaccines targeting oxycodone and hydrocodone | | | | |
| --- | --- | --- | --- | --- |
| References | Drugs | Animal | Main Findings | Vaccine types |
| (Pravetoni et al., 2014a) | Oxycodone | Male Rats | - Reduced acquisition of intravenous (i.v.) oxycodone self-administration. - Attenuated the reinforcing effects of oxycodone. | A vaccine comprising a hapten (OXY) conjugated to the carrier protein keyhole limpet hemocyanin (OXY-KLH) |
| (Pravetoni et al., 2012) | Oxycodone | Rats | - Three vaccinations were administered on days 0, 21, and 42, with blood collected 7 to 10 days after the final immunization. - Produced high-titer antibodies specific to oxycodone (OXY) and its metabolite oxymorphone. - Significant reduction in OXY distribution to the brain. - Reduced oxycodone-induced antinociception. | OXY(Gly)_4_-KLH, [OXY conjugated to carrier proteins, such as bovine serum albumin (BSA) or keyhole limpet hemocyanin (KLH). Two different linkers, tetraglycine (Gly)_4_ and hemisuccinate (HS), are evaluated for attachment to the 6-position of OXY] |
| (Raleigh et al., 2017) | Oxycodone | Rats | - Effectively blocked the antinociceptive effects of oxycodone. - Protective effect against oxycodone-induced respiratory depression. - Did not interfere with naloxone's effectiveness. - High specificity for oxycodone and its active metabolite oxymorphone. - Extended-release naltrexone administration did not affect OXY-KLH vaccine immunogenicity. - Continuous morphine infusion during vaccination did not impair OXY-dKLH immunogenicity. | Oxycodone conjugated to keyhole limpet hemocyanin subunit dimer (OXY-dKLH) adsorbed to alum. |
| (Pravetoni et al., 2013) | Oxycodone | Rats | - Reduced drug distribution to the brain and blunted analgesia for both oxycodone and hydrocodone. - Significantly increased drug binding in serum. Consistent reduction in oxycodone effects. - Serum antibodies generated by the vaccine showed cross-reactivity and recognition of both oxycodone and hydrocodone. | 6OXY(Gly)4–KLH |
| (Pravetoni et al., 2014b) | Oxycodone | Mice and Rats | - Effectively prevented oxycodone-induced antinociception. - 6OXY conjugated to tetanus toxoid (TT) or GMP-grade KLH dimer (dKLH) exhibited equivalent effectiveness to 6OXY conjugated to nKLH decamer. - The 6OXY hapten conjugated to a TT-derived peptide failed to prevent oxycodone-induced antinociception. | Oxycodone derivatized at the C6 position (6OXY) conjugated to different carrier proteins such as native keyhole limpet hemocyanin (nKLH), tetanus toxoid (TT), and a GMP grade KLH dimer (dKLH) |
| (Laudenbach et al., 2018) | Oxycodone | Mice | - Blockage of IL-4 signaling increased vaccine efficacy in blocking oxycodone distribution to the brain. - Co-administration of the oxycodone vaccine (OXY-KLH) with an anti-IL-4 monoclonal antibody (αIL-4) significantly increased vaccine efficacy against oxycodone. - Alleviate doxycodone-induced respiratory depression and bradycardia. | OXY-KLH |
| (Taylor et al., 2014) | Oxycodone | Mice | - 6OXY-KLH was more effective than 8HYDROC-KLH in preventing oxycodone distribution to the brain. - Vaccination with 6OXY-KLH selectively induced 6OXY-specific B cell phenotypes within the polyclonal response. - The detection of activated hapten-specific B cells provided an earlier indication of vaccine success or failure than serum antibody responses. | 6OXY-KLH, 8HYDROC-KLH |
| (Robinson et al., 2019) | Oxycodone | Mice | - Alum was more effective than MF59 in promoting the early expansion of hapten-specific B cells and the production of oxycodone-specific antibodies. - Alum outperformed MF59 in blocking oxycodone distribution to the brain and reducing oxycodone-induced motor activity. - Alum was more effective in generating early B cell subsets, including germinal center (GC) B cells, and promoting early Tfh and GC-Tfh cell responses. | OXY-KLH |
| (Nguyen et al., 2018) | Oxycodone | Male Wistar Rats | - Half an hour after injection, brain oxycodone concentrations in vaccinated rats were 50% lower compared to those in TT rats. - Oxy-TT rats exhibited lower sensitivity to oxycodone reinforcement, with only 58% acquiring self-administration at a lower dose. - Decreased sensitivity to oxycodone-induced antinociception in a dose-dependent manner. | Oxy-TT |
| (Crouse et al., 2023) | Oxycodone | Mice | - Mice were immunized with the vaccine on days 0, 14, and 28, with or without IL-4 depletion, and serum was collected on day 34 to analyze oxycodone-specific and total IgE concentrations. - IL-4 depletion does not induce class switching to IgE, and no oxycodone-specific IgE is detected after OXY-sKLH immunization. - IgG2a Abs demonstrate equivalent efficacy to IgG1 Abs against the oxycodone challenge. - Preimmunization production of IL-4 from T cells correlates with postimmunization antibody titers and vaccine efficacy. | OXY-sKLH |
| (Laudenbach et al., 2015) | Oxycodone | Mice | - Mice received subcutaneous immunizations on days 0, 14, and 28, and in those analyzed by partial splenectomy on day 35, 6OXY-specific serum IgG titers were measured. - Higher frequencies of 6OXY-specific B cells, both before and after immunization, were correlated with greater oxycodone-specific serum Ab titers and efficacy in blocking oxycodone distribution to the brain. - The effectiveness of the 6OXY vaccine and the activation of 6OXY-specific B cells were strongly correlated with the size of the CD4+ T cell population. - Depletion of CD4+ T cells and analysis of carrier-specific CD4+ T cells indicated their interrelation with hapten-specific B cells and contribution to vaccine efficacy. - The frequency of 6OXY-specific B cell subsets across various tissues, including spleen, blood, and lymph nodes, was consistent. | 6OXY-KLH |
| (Gradinati et al., 2020) | Oxycodone | Mice and Rats | - Polymer-based delivery of the anti-oxycodone vaccine (OXY-KLH) was as effective as or more effective than traditional aluminum adjuvant in inducing oxycodone-specific antibodies. - A single injection of OXY-sKLH formulated in three polymer thermo sensitive (PTS) gels with different release kinetics provided equivalent or superior protection against oxycodone compared to the standard protocol involving multiple injections of OXY-sKLH and alum adjuvant. - The triphasic polymer formulation was particularly effective in blocking oxycodone-induced effects, including antinociception, respiratory depression, and bradycardia. | OXY-KLH and OXY-sKLH |
| (Hamid et al., 2022) | Oxycodone | Rats | - Administered intramuscularly on days 1, 15, 29, and 43, resulting in significantly higher oxycodone-specific IgG titers in vaccinated rats on day 50. - Induced a dose-dependent and long-lasting immune response. - No vaccine-related toxicity was observed, and no adverse effects were evident before or after oxycodone administration. | Oxy(Gly)4-sKLH |
| (Raleigh et al., 2021) | Oxycodone | Rats | - Antibodies induced by the Oxy(Gly)4-sKLH vaccine persisted for 2-3 months following the final vaccination. - Vaccination selectively reduced oxycodone-induced antinociception without altering the effects of methadone, fentanyl, or naloxone. - Significantly diminished the reinforcing effects of oxycodone in an intravenous self-administration model. - No evidence of toxicity. | Oxy(Gly)4-sKLH |
| (Huseby Kelcher et al., 2021) | Oxycodone | Mice | - Depletion of macrophages and granulocytes did not affect the efficacy of the lead vaccine against oxycodone. - Higher doses of F(ab)2 fragments were required to achieve efficacy comparable to lower doses of intact mAbs. - FcRn was found to play a critical role in maintaining efficacious levels of serum antibodies upon active vaccination, contributing to the optimal pharmacokinetic performance of vaccines and mAbs. | OXY-KLH and OXYsKLH |
| (Kimishima et al., 2017) | Oxycodone, Hydrocodone | Mice | - Effectively reduced the analgesic efficacy of oxycodone and hydrocodone. - Retained their efficacy in reducing opioid antinociceptive effects, even at higher doses. - Increased survival rates when subjected to lethal doses of oxycodone and hydrocodone. | Oxycodone-TT (Oxy-TT) and Hydrocodone-TT (Hydro-TT) |
| (Raleigh et al., 2018) | Heroin, Oxycodone | Rats | - M-KLH reduced total opioid distribution to the brain, particularly at lower heroin doses. - M-KLH was more effective against respiratory depression induced by lower heroin doses. - The efficacy of M-KLH was highly dependent on heroin doses and less effective at higher doses. - OXY-KLH effectively attenuated oxycodone-induced antinociception. - The efficacy of OXY-KLH was dose- and route-dependent, with better results observed following subcutaneous administration than intravenous administration. | A heroin vaccine [morphine hapten conjugated to keyhole limpet hemocyanin (M-KLH)]Oxycodone vaccine [oxycodone hapten conjugated to keyhole limpet hemocyanin (OXY-KLH)] |
| (Baruffaldi et al., 2018) | Heroin, Oxycodone | Mice | - Morphine-based haptens conjugated to carrier proteins (sKLH and EcoCRM) are equally effective in reducing heroin-induced effects. - Various carrier proteins, including EcoCRM, rTTHc, KLH, TT, and CRM197, demonstrate equal effectiveness in inducing hapten-specific B cell expansion and reducing oxycodone distribution to the brain. | OXY(Gly)4-KLH, OXY(Gly)4-SH-KLH, M(Gly)4OH-KLH, M-sKLH and M-EcoCRM |
| (Crouse et al., 2020) | Oxycodone, Fentanyl | Mice | - Administered intramuscularly or subcutaneously on days 0, 14, and 28, with blood collected on days 14 and 34 for antibody analysis. - Blocking IL-4 improved the efficacy of anti-opioid vaccines, targeting oxycodone and fentanyl. - IL-4 depletion increased oxycodone-specific IgG titers in both male and female mice. - IL-4 depletion resulted in increased germinal center formation in secondary lymphoid organs after vaccination. | OXY-KLH, OXYsKLH, F-sKLH, and F-CRM |
