# Supplementary material for: The development of opioid vaccines as a novel strategy for the treatment of opioid use disorder and overdose prevention
Source: Int J Neuropsychopharmacol. 2025 Jan 20;28(2):pyaf005. doi: 10.1093/ijnp/pyaf005 (PMC11792077; doi:10.1093/ijnp/pyaf005)
Supplement: pyaf005_suppl_Supplementary_Table_S3 [file pyaf005_suppl_supplementary_table_s3.pdf]

Supplementary Table 3. Vaccines targeting fentanyl, synthetic fentanyl analogs, and other synthetic opioids

References	Drugs	Animal	Main Findings	Vaccine types
(Raleigh et al., 2019)	Fentanyl	Mice and rats	• Reduced fentanyl-induced antinociception.<br>• Decreased brain fentanyl concentrations.<br>• Attenuated fentanyl-induced antinociception and respiratory depression.	Fentanyl-based hapten (F) conjugated to a carrier protein, either keyhole limpet hemocyanin (KLH) or GMP-grade subunit KLH (sKLH)
(Haile et al., 2022)	Fentanyl	Male and female rats	• A significant rise in anti-FEN IgG antibody levels over a period of ten weeks effectively inhibited FEN-induced antinociception. Lower brain FEN levels in vaccinated rats.<br>• Prevented FEN-induced decreases in oxygen saturation, heart rate, and activity.	A Fentanyl (FEN) vaccine referred to as "FEN-CRM+dmLT." This vaccine is a conjugate formulation, with CRM197 as the carrier protein and dmLT (double-mutant heat-labile toxin) as the adjuvant.
(Townsend et al., 2019)	Fentanyl	Male and female rats	• Decreased the reinforcing effects of fentanyl, leading to a substantial decrease in fentanyl-vs.-food choice behavior.<br>• Reduction in fentanyl reinforcement for 15 weeks, highlighting the sustained efficacy of the vaccine.<br>• Prevented withdrawal-associated increases in fentanyl preference over food.<br>• Decreased fentanyl self-administration.<br>• Comparable effectiveness to the naltrexone.	Fentanyl-tetanus toxoid conjugate (Fent-TT)
(Bremer et al., 2016)	Fentanyl	Mice	• Significant protection from potentially lethal doses of fentanyl analogues.<br>• Exhibited a significant reduction in fentanyl concentrations in the brain.	Fent-TT
(Tenney et al., 2019)	Fentanyl	Male rhesus monkeys	• Reduced fentanyl's rate-suppression potency by approximately 10-fold.<br>• Decreased fentanyl's antinociceptive potency by roughly 25-fold.<br>• Demonstrated comparable effectiveness to an acute dose of 0.032 mg/kg naltrexone.<br>• Exhibited selectivity for fentanyl over oxycodone.<br>• The antibody immune response showed an affinity of ~3 nM for fentanyl.<br>•	Fent-TT
(Stone et al., 2021)	Fentanyl	Mice	• Mucosal booster immunizations, including sublingual dmLT or intranasal LTA1, resulted in high levels of anti-fentanyl immunity.<br>• Additional mucosal booster immunizations with dmLT or LTA1 further increased the magnitude of anti-fentanyl immunity following an intramuscular series.	FEN-TT vaccine adjuvanted with LTA1 (intranasal) or dmLT (sublingual)

			• Protection from fentanyl-induced antinociception (pain relief) and brain tissue distribution.<br>• IgA antibodies induced by mucosal booster immunizations showed a significant correlation with protection from fentanyl challenge.	
(Townsend et al., 2021)	Fentanyl	Rhesus monkeys	• Buprenorphine significantly reduced fentanyl choice and increased food choice.<br>• Vaccination eliminated fentanyl choice and increased food choice in four out of five monkeys.<br>• A transient and less pronounced vaccine effect was observed in the fifth monkey.<br>• Fentanyl-specific antibody concentrations peaked at approximately 50 µg/mL after the third vaccination.<br>• Anti-fentanyl antibody affinity improved to a sustained low nanomolar level.	A fentanyl-CRM197 conjugate vaccine
(Barrientos et al., 2020)	Fentanyl	Mice	• Mice were immunized intramuscularly at weeks 0, 3, 6, and 14, with blood samples collected at weeks 0, 3, 6, 9, 14, and 16, showing higher mean endpoint titers in immunized mice compared to unimmunized mice at week 16.<br>• Induced high and robust antibody endpoint titers (>10^6) against the fentanyl hapten.<br>• No cross-reactivity was observed with naloxone, naltrexone, methadone, or buprenorphine.<br>• Effectively blocked the antinociceptive effects of fentanyl.	TT–para-AmFenHap
(Robinson et al., 2020)	Fentanyl	Mice and rats	• Prophylactic vaccination reduced fentanyl-induced antinociception, respiratory depression, and bradycardia in mice and rats.<br>• Reduced fentanyl intravenous self-administration in rats.<br>• Did not interfere with the pharmacological activity of oxycodone, heroin, methadone, or naloxone.	F1–sKLH, F1–CRM1, F1–CRM2
(Miller et al., 2023)	Fentanyl	Mice	• Significantly increased protection against fentanyl-induced bradycardia.<br>• Alum + INI-4001 resulted in significantly increased serum fentanyl concentrations and decreased brain fentanyl concentrations after fentanyl challenge.	F1-CRM+alum+INI-4001
(Wang et al., 2020)	Fentanyl	Mice	• The opsonization effect of anti-Gal antibodies effectively enhanced the immune response, minimizing the need for additional adjuvants.<br>• The vaccine's effectiveness was verified using in vivo antinociception assays, revealing 3 to 5-fold escalation in the fentanyl dose required to elicit the drug's effects.<br>• Fentanyl blood-brain distribution studies indicated a sequestering effect by the vaccine, supporting its potential in preventing fentanyl from entering the brain.	Double-conjugate fentanyl vaccine leveraging preformed anti-Gal antibodies [fentanyl-(α-Gal)-OVA]
(Eubanks et al., 2021)	Carfentanil	Mice	• Complete blockade of carfentanil-induced respiratory depression.<br>• Attenuation of carfentanil-induced antinociception.<br>• Significantly lower levels of carfentanil in the brain.	Conjugate vaccines using haptens (Carfen-ester-TT and Carfen-p-phenyl-TT) that were chemically synthesized and then conjugated to the carrier protein tetanus toxoid (TT)

(Park et al., 2023)	U-47700 [3,4-dichloro-N-((1R,2R)-2-(dimethyl amino)cyc lohexyl)-N-methyl benzamide]	Mice	• The U-47700 vaccine altered drug biodistribution, leading to substantial retention of the drug within the bloodstream and preventing its unhindered diffusion into the brain.<br>• Elevated antibody titers in rodents with sub-micromolar affinity to U-47700.<br>• Blocked the drug's penetration into the blood-brain barrier, as demonstrated by antinociception and drug biodistribution studies.	U-47700-KLH
(Lee et al., 2022)	Benzimid azole-derived NPS opioids (BNO)	Mice	• Injected intraperitoneally into mice on weeks 0, 2, and 4, and blood samples collected on weeks 3 and 5 showed robust antibody titers, with a threefold increase after the second boost.<br>• Produce high-titer antibodies with nanomolar affinity targeting multiple benzimidazole-derived NPS opioids (BNO).<br>• Antibodies mitigated the psychoactive and physiological effects of BNO exposure, demonstrated through antinociception, whole-body plethysmography, and blood-brain biodistribution studies.<br>• Documented pharmacokinetics of BNO drugs	BNO-KLH and BNO-BSA
(Crouse et al., 2021)	Fentanyl, Oxycodon e	Male mice and rats	• Housing conditions do not impact the efficacy of fentanyl vaccines in mice.<br>• Both conventional and specific pathogen-free (SPF, sterile barrier maintained) housed mice exhibit a significant reduction in fentanyl-induced analgesia and brain distribution.<br>• Vaccinated rats show protection against oxycodone-induced respiratory depression and bradycardia under both housing conditions.	OXY-sKLH and F-sKLH
(Baehr et al., 2022)	Fentanyl, Alfentanil, Sufentanil , Acetylfent anyl	Rats	• Reduced antinociception, respiratory depression, and bradycardia induced by fentanyl, sufentanil, and acetylfentanyl.<br>• Significantly lowered fentanyl levels in the brains of rats exposed to lethal doses.	F1–CRM
