# Supplementary material for: The development of opioid vaccines as a novel strategy for the treatment of opioid use disorder and overdose prevention
Source: Int J Neuropsychopharmacol. 2025 Jan 20;28(2):pyaf005. doi: 10.1093/ijnp/pyaf005 (PMC11792077; doi:10.1093/ijnp/pyaf005)
Supplement: pyaf005_suppl_Supplementary_Table_S4 [file pyaf005_suppl_supplementary_table_s4.pdf]

Supplementary Table 4. Bivalent opioid vaccines

References	Drugs	Animal	Main Findings	Vaccine types
(Hwang et al., 2018a)	Fentanyl and Heroin	Mice	• The admixture vaccine outperformed individual vaccines in antinociception.<br>• The admixture vaccine significantly outperformed individual vaccines in behavioral models.	Admixture vaccine and tetanus toxoid (TT), and then combining two individual immunoconjugate vaccines to target heroin and fentanyl; Fent-TT + Her-TT
(Hwang et al., 2018b)	Heroin and Fentanyl	Mice	• Drug retention in serum and reduced distribution in the brain were observed in rodents immunized with the admixture vaccine after intravenous administration of heroin mixed with fentanyl.<br>• Generated potent antibodies against both heroin and fentanyl.<br>• Effectively reduced heroin and fentanyl nociception effects.	Admixture vaccine, Her- KLH and Fent- KLH
(Blake et al., 2020)	Heroin and Fentanyl	Mice and cynomolgus monkeys	• Non-human primates vaccinated with a heroin/fentanyl combination vaccine demonstrated potent antibody responses.<br>• CRM showed superior performance over traditional carrier protein TT, providing a cost-effective and efficient alternative.<br>• Lyophilized vaccine formulations stored at room temperature maintained efficacy for up to 1 year.	Her-TT Vaccine, Her-CRM & fentanyl-CRM
(Barrientos et al., 2021)	Heroin and Fentanyl	Mice	• Polyclonal sera from the bivalent vaccine group exhibited strong binding to heroin, 6-acetylmorphine (6-AM), morphine, and fentanyl.<br>• Serum from bivalent vaccine-immunized mice effectively sequestered heroin, 6-AM, morphine, and fentanyl.<br>• Significant binding against a mixture of heroin + 9% fentanyl was observed.<br>• Immunized mice demonstrated protection against thermal antinociception induced by heroin, fentanyl, and a heroin + 9% fentanyl mixture.	A heroin hapten (6-AmHap), and a fentanyl hapten (para-AmFenHap), conjugated to tetanus toxoid (TT)
(Townsend et al., 2020)	Heroin and Fentanyl	Rats	• Generated high-affinity antibodies against both opioids.<br>• Significantly decreased the antinociceptive potency of fentanyl, heroin, and fentanyl/heroin mixture.<br>• Did not impact the antinociceptive effectiveness of methadone.<br>• Did not significantly impact the self-administration of the fentanyl/heroin mixture in a drug-vs-food choice procedure.	Heroin-CRM and Fentanyl-CRM
(Baruffaldi et al., 2019)	Heroin and Oxycodone	Rats	• Vaccine efficacy was influenced by dose, with 60 µg of immunogen identified as the most effective dose for OXY-sKLH.<br>• Immunized intramuscularly on days 0, 21, 42, and 63, with serum collected on day 70.<br>• Co-administration of OXY-sKLH and M-sKLH in a bivalent vaccine formulation enhanced serum antibody titers without compromising individual vaccine efficacy.	OXY-sKLH and M-sKLH

(Pravetoni et al., 2012)	Morphine and Oxycodone	Rats	• Vaccinated on days 0, 14, and 28, with blood collected on day 35 to measure serum antibody titers.<br>• Rats immunized solely with M-KLH exhibited elevated antibody titers specific to heroin, 6-monoacetylmorphine (6-MAM), and morphine.<br>• Immunization using OXY-KLH resulted in heightened antibody titers against oxycodone and oxymorphone.<br>• The bivalent vaccine consistently induced robust antibody responses against both antigens.<br>• The efficacy of the vaccines showed a direct correlation with serum antibody titers.	OXY-KLH vaccine, M-KLH vaccine, Bivalent M-KLH + OXY-KLH vaccine
(Natori et al., 2019)	Heroin and Fentanyl	Female BALB/cByJ mice	• The best-performing vaccines showed comparable protection against heroin compared to the benchmark heroin vaccine.<br>• The HF-7 conjugate vaccine demonstrated a balanced immune response against heroin and some efficacy against fentanyl in a heroin mixture.	HF-1, HF-2, HF-3, HF-4, HF-5, HF-6, HF-7, HF-8, and HF-9
(Park et al., 2021)	Heroin and Fentanyl	Female BALB/c mice	• The dual hapten vaccine produced antibodies with nanomolar affinities, effectively binding to fentanyl-BSA and dual-BSA in ELISA assays.<br>• The dual hapten vaccine demonstrated protective effects against both heroin and fentanyl exposure in tail flick and hot plate tests.<br>• Significantly higher ED50 values were observed for 10% fentanyl in heroin.<br>• The dual hapten vaccine showed comparable or improved efficacy compared to positive control vaccines targeting individual heroin or fentanyl haptens.	Heroin- CRM, Fentanyl- CRM and dual-CRM
(Crouse et al., 2022)	Carfentanil and Fentanyl	Rats	• Rats were immunized with conjugate vaccines containing carfentanil-based, fentanyl-based haptens, or their combination in bivalent formulations, then challenged with the opioids.<br>• Protection against carfentanil-induced antinociception, respiratory depression, and bradycardia.<br>• Bivalent vaccination strategies using heterologous prime/boost or co-administration regimens protected against both carfentanil and fentanyl.	F1-CRM (fent-CRM) + F13-CRM (Carf-CRM)
